# Effects of social support, hope and resilience on quality of life among Chinese bladder cancer patients: a cross-sectional study

**DOI:** 10.1186/s12955-016-0481-z

**Published:** 2016-05-06

**Authors:** Meng-Yao Li, Yi-Long Yang, Li Liu, Lie Wang

**Affiliations:** Department of Social Medicine, School of Public Health, China Medical University, No.77 Puhe Road, Shenyang North New Area, Shenyang, 110122 PR China

**Keywords:** Bladder cancer, Psycho-social factors, Quality of life, Social support, Hope, Resilience

## Abstract

**Background:**

Improvement of quality of life has been one of goals in health care for people living with bladder cancer. Meanwhile, positive psycho-social variables in oncology field have increasingly received attention. However, the assessment of quality of life of bladder cancer patients and the integrative effects of positive psycho-social variables has limited reporting. The aim of this study was to assess quality of life as well as the integrative effects of social support, hope and resilience on quality of life among Chinese bladder cancer patients.

**Methods:**

A cross-sectional study was conducted at the First Hospital of China Medical University in Liaoning Province, China. A total of 365 bladder cancer patients eligible for this study completed questionnaires on demographic variables, FACT-BL, Perceived Social Support Scale, Adult Hope Scale, and Resilience Scale-14 during July 2013 to July 2014.

**Results:**

The average score of FACT-BL was 87.60 ± 16.27 (Mean ± SD). Hierarchical regression analyses indicated that social support, hope and resilience as a whole accounted for 30.3 % variance of quality of life. Under standardized estimate (β) sequence, social support, hope and resilience significantly and positively associated with quality of life, respectively.

**Conclusions:**

Quality of life for bladder cancer patients was at a low level in China, which should receive more attention in Chinese medical institutions. More importantly, efforts to increase social support, hope and resilience might be useful to support the quality of life among Chinese bladder cancer patients.

## Background

Urogenital malignant tumor, as a serious and potentially life-threatening illness, is a major public health problem in the world. At present, morbidity of bladder cancer is highest in urogenital malignant tumor, ranked eighth in the morbidity of Chinese malignant tumor, and accounted for 2.50 % of the morbidity of malignant Chinese tumor [1]. In 2008, the morbidity of bladder cancer in China was 7.49/100,000, and the standardized morbidity of world population was 4.53/100,000 [1]. Morbidity of Chinese male bladder cancer patients is 3.3 times higher than that of females. In recent decades, morbidity and mortality of Chinese bladder cancer have increased year by year.

Improvement of quality of life (QOL) has been one of goals in health care for people living with bladder cancer. Besides the general impacts of demographic and clinical differences, psychological and social states also influence the QOL. Researchers increasingly recognize the value of considering how to improve the QOL of patients with bladder cancer and prolong the survival length [2]. On the basis of the literature review, external factor (social support) and internal factors (hope and resilience) were key research points in this field.

Social support is generally defined as perceived comfort, caring, assistance and esteem one individual receives from others [3]. The presence or absence of social support may be an important factor influencing the development and progression of cancer [4, 5]. Social support leads the individuals to believing that themselves are concerned and accepted, in the meantime, there is someone who appreciates and takes care of them [6]. The presence of supportive interpersonal relationships has the potential to influence well-being in cancer survivorship [7], and it is also shown to be significant mediators of optimistic and positive affect [8]. Hope is defined as one’s belief in the capability to achieve goals, particularly in situations where one can influence outcomes through the use of personal abilities or strengths [9]. In the classic model, hope is composed of six dimensions: cognitive, temporal, contextual, affective, affiliative and behavioural dimensions [10]. But Snyder et al. defined hope as a cognitive set that is composed of a reciprocally derived sense of successful agency (goal-directed determination) and pathways (planning of ways to meet goals), an individual-differences measure is developed [11]. For patients with cancer, hope is regarded as one of the most important and effective coping style in fighting against the cancer during treatment [12]. Resilience has been defined as a particular trajectory or mechanism of positive adaptation that changes over time and protects against psychological distress [13]. It is an individual’s capacity to maintain psychological and physical well-being in the face of adversity situation [14], including mental health, functional capacity, and social competence and even traumatic events [15]. Resilience is associated with lower-level distress, better adjustment, and better QOL among cancer patients [14, 16], and helps to relieve cancer related psychological problems [17].

While positive resources are getting attention in oncology field, there are still few studies exploring the integrative effects of two aspects (external and internal factors) on QOL of patients with bladder cancer. Similarly, QOL has been extensively studied among cancer patients, few studies used a relatively large sample (*n* >300) to assess QOL and the associated factors in Chinese bladder cancer patients. In light of these above concerns, there were two main aims: 1) the present study was designed to assess the QOL for bladder cancer patients, and to clarify the associated factors; 2) this study designed to investigate the integrative effects of positive psycho-social variables on QOL after adjusting for the demographic and clinical variables.

## Methods

### Study design and study sample

A cross-sectional study was carried out from July 2013 to July 2014. All participants came from the First Hospital of China Medical University, and underwent surgical treatments. Inclusion criteria in this study were that participants (1) were at least 18 years old, (2) had primary school diploma or above, (3) with pathological diagnosis of bladder cancer, (4) were able to communicate in Chinese language well enough to answer the questionnaires, (5) had clear consciousness and cognition (be able to accurately answer questions on persons, place, and time within 30 s). Exclusion criteria were the following: (1) patients had a history of psychiatric problems (depression, anxiety, and other psychiatric disorders) before cancer diagnose, (2) patients had intellectual impairments, (3) patients had other active cancers. After obtaining the informed consent to conduct this survey, self-administered questionnaires were distributed to participants. There were strict quality control measures to avoid possible bias. Each patient was given the questionnaire to complete in a private place within one week after surgery. Of the 424 bladder cancer patients who met the inclusion criteria, 59 patients were excluded because they declined to participate or the missing values exceeding 30 % in the questionnaire. Finally, the effective response rate was 86.08 %.

### Measurement of QOL

QOL was measured with FACT-BL. The Functional Assessment of Cancer Therapy- general form (FACT-G) was developed in the early 90s [18]. It is comprised of 27 questions, and consisted of four dimensions: physical well-being (PWB), social/familial well-being (SWB), emotional well-being (EWB) and functional well-being (FWB). It has 27 questions in total, and has been translated into more than 30 languages. FACT-BL, as a bladder cancer-specific instrument, has been validated in previous researches [18, 19]. It is comprised of FACT-G and 12 bladder cancer specific questions (BSS). Each item had five responses ranging from 0 “not at all” to 4 “very much”. The Chinese version of the FACT-BL (version 4) had been used in Chinese studies, and it had demonstrated adequate reliability and validity [20]. In the present study, the Cronbach’s alpha coefficients of PWB, SWB, EWB, FWB, BSS and the total scale were 0.866, 0.918, 0.772, 0.874, 0.795 and 0.836, respectively. The total score of the total scale was calculated to get a composite QOL value in this research, with higher scores indicating higher levels of QOL.

### Measurement of social support

Social support was assessed using the 12-item version Perceived Social Support Scale (PSSS), which developed by Zimet et al. [21]. This scale was divided into three domains: family support, friends support and significant others. In this study, the total score of social support was used. The item is 7-point rating ranging from one “very strongly disagree” to “very strongly agree”. The higher total score reflects better PSSS. This reliable and valid measure also shows good psychometric properties with cancer patients. The Chinese version had been used in Chinese researches, and it had demonstrated adequate reliability and validity [22]. The Cronbach’s alpha coefficient was 0.968 in this research.

### Measurement of hope

The 12-item Adult Hope Scale (AHS) was used to assess patients’ trait levels of hope [11]. The scale includes two dimensions: agency and pathway, each of them contains four items. The remaining four items are fillers. In Chinese version, each item was answered using a 4-point Likert-type scale ranging from “strongly disagree” to “strongly agree” [23]. The hope scale reflects the sum of the agency and pathways items, in which a high score indicates a higher level of hope. The Chinese version had been widely used in Chinese researches, and had been yield accurate results [23]. In this present study, Cronbach’s alpha coefficient of total scale was 0.846.

### Measurement of resilience

The Resilience Scale-14 (RS) was widely used in resilience research [24]. The item is answered using a 7-point Likert-type scale ranging from “strongly disagree” to “strongly agree” [25]. The total score of the total scale was calculated to get a composite resilience value with higher scores indicating higher levels of resilience. The Chinese version had been used in previous researches, and the reliability and validity had been repeatedly confirmed [24, 25]. In this present study, the Cronbach’s alpha coefficient of total scale was 0.953.

### Demographic and clinical characteristics

Demographic characteristics (age, education level, marital status, chronic diseases status, smoking/drinking status, physical activity and familial inheritance) and clinical characteristics (time since first cancer diagnosis, perfusion chemotherapy, cancer stages, histopathological grading and pathologic features) were obtained in this study. Age was divided into “18–55 years”, “56–65 years”, “66–75 years” and “over 75 years”. Education level was categorized as “primary/middle school”, “high/secondary school” and “junior college and over”. Marital status was categorized into two groups, as “single/divorced/separated/widow” and “married/cohabitation”. Time since first cancer diagnosis was categorized into “≤1 month” and “>1 month”. Cancer stages were divided into “I” and “II+III”. Histopathological grading was divided into “G1” and “G2+G3”. Pathologic features were categorized into “urothelial carcinoma”, “adenocarcinoma” and “squamous cell carcinoma”. Others were divided into “yes” or “no” two groups.

### Statistical analysis

All analyses were performed with the SPSS 17.0 program. And all statistical tests were two-sided (α = 0.05). Descriptive statistics of the demographic, clinical and study variables were indicated with mean, standard deviation (SD), number (n) and percentage (%) as appropriate. Study variables were compared between age groups, education level groups and pathologic features by one-way ANOVA analyses. T-tests were performed to examine the differences in continuous variables between gender, chronic diseases, smoke/drink habit, physical activity, familial inheritance, time since first cancer diagnosis, perfusion chemotherapy, cancer stages, and histopathological grading. When one-way ANOVAs were found to be significant, least-significant-difference tests (LSDs) were done to perform multiple comparisons. Correlations between QOL and positive resources were examined by Pearson’s correlation. If the correlation between two variables was more than 0.05, these variables were adjusted in the multivariate analysis. Furthermore, two measures, tolerance and variance inflation factor, were used to check for multicollinearity. Hierarchical regression analyses were conducted to indicate the effects of influence factors on QOL. In step 1 of the hierarchical linear regression analyses, the control variables (age, education level and physical activity) were used as predictors. Social support, hope and resilience were entered into step 2. Standardized estimate (β), F, R^2^, Adjusted R^2^, and R^2^-change (ΔR^2^) for each step were provided in the regression models.

## Results

### Descriptive statistics

Demographic and clinical characteristics of participants were shown in Table 1. The participants (*n* = 365) were in the age range of 18–90, and mean (SD) age of participates was 63.76(11.45) years old. 80.27 % of these participates were males. Nearly 90.41 % of the participants were married or cohabitated, and 45.48 % of them exercised regularly. 82.2 % of the participants were newly-diagnosed, and 57 % accepted the perfusion chemotherapy after surgery.

**Table 1:** Descriptive statistics for QOL and its components. (N = 365)

Variable	*N* (%)	FACT-G	FACT-BL	PWB	SWB	EWB	FWB	BSS
		Mean ± SD	Mean ± SD	Mean ± SD	Mean ± SD	Mean ± SD	Mean ± SD	Mean ± SD
Gender								
Male	293 (80.27)	68.13 ± 16.46	87.93 ± 16.62	19.33 ± 5.42	17.47 ± 6.84	15.33 ± 4.65	15.99 ± 6.12	19.80 ± 7.37
Female	72 (19.73)	67.96 ± 15.76	86.26 ± 14.82	18.64 ± 6.07	18.10 ± 6.83	15.70 ± 4.39	15.52 ± 6.07	18.30 ± 6.85
Age								
18–55	74 (20.27)	68.30 ± 16.13*	88.22 ± 18.13	19.74 ± 5.55	17.21 ± 7.48	15.09 ± 4.88	16.26 ± 7.04	19.92 ± 8.27
56–65	125 (34.25)	69.35 ± 16.46*	88.00 ± 15.78	19.71 ± 5.31	17.62 ± 6.65	15.99 ± 4.27	16.03 ± 6.02	18.65 ± 6.66
66–75	115 (31.51)	69.36 ± 15.77*	88.92 ± 15.36*	19.04 ± 5.42	18.52 ± 6.70	15.54 ± 4.58	16.25 ± 5.17	19.56 ± 7.34
>75	51 (13.97)	61.85 ± 16.43*	82.73 ± 16.25*	17.53 ± 6.23	15.98 ± 6.45	14.09 ± 4.80	14.26 ± 6.73	20.88 ± 7.03
Education level								
Primary/Middle school	210 (57.53)	66.00 ± 15.55*	85.47 ± 15.92**	19.12 ± 5.42	16.87 ± 6.91*	14.97 ± 4.40*	15.03 ± 5.99*	19.48 ± 7.39
High or secondary school	85 (23.29)	68.71 ± 14.95	88.05 ± 14.45**	19.59 ± 4.93	17.53 ± 6.42*	15.49 ± 4.29	16.10 ± 5.41*	19.34 ± 6.71
Junior College and over	70 (19.18)	73.63 ± 18.81*	93.43 ± 18.09**	18.96 ± 6.63	19.83 ± 6.71*	16.59 ± 5.33*	18.25 ± 6.67*	19.80 ± 7.71
Marital status								
Single/Divorced/Separated/Widow	35 (9.59)	68.16 ± 14.46	88.46 ± 15.46	19.25 ± 5.24	17.65 ± 6.17	15.97 ± 4.80	15.29 ± 5.83	20.30 ± 7.71
Married/Cohabitation	330 (90.41)	68.09 ± 16.51	87.51 ± 16.38	19.19 ± 5.60	17.58 ± 6.91	15.34 ± 4.58	15.96 ± 6.14	19.42 ± 7.25
Chronic diseases								
No	194 (53.15)	68.20 ± 16.23	87.06 ± 16.59	20.09 ± 4.93**	16.97 ± 7.22	15.83 ± 4.34	15.31 ± 6.32	18.86 ± 6.94
Yes	171 (46.85)	67.97 ± 16.44	88.21 ± 15.94	18.19 ± 6.05**	18.30 ± 6.32	14.92 ± 4.84	16.56 ± 5.80	20.24 ± 7.61
Smok								
No	161 (44.11)	67.88 ± 16.21	87.16 ± 16.09	19.30 ± 5.55	17.22 ± 7.12	15.60 ± 4.36	15.78 ± 5.89	19.28 ± 7.06
Yes	204 (55.89)	68.26 ± 16.42	87.95 ± 16.45	19.12 ± 5.57	17.89 ± 6.60	15.25 ± 4.78	15.99 ± 6.29	19.69 ± 7.47
Drink								
No	221 (60.54)	67.58 ± 16.10	86.81 ± 16.05	19.29 ± 5.50	17.28 ± 6.97	15.33 ± 4.77	15.67 ± 6.12	19.23 ± 7.51
Yes	144 (39.45)	68.88 ± 16.65	88.81 ± 16.60	19.06 ± 5.65	18.08 ± 6.61	15.51 ± 4.32	16.24 ± 6.10	19.93 ± 6.93
Physical activity								
No	199 (54.52)	66.31 ± 15.93*	85.65 ± 16.04*	18.72 ± 5.62	16.83 ± 6.56*	15.08 ± 4.62	15.67 ± 5.74	19.34 ± 7.07
Yes	166 (45.48)	70.23 ± 16.54*	89.94 ± 16.30*	19.78 ± 5.43	18.50 ± 7.06*	15.78 ± 4.55	16.18 ± 6.53	19.70 ± 7.56
Familial inheritance								
No	304 (83.29)	68.14 ± 16.67	87.46 ± 16.36	19.35 ± 5.61	17.41 ± 6.82	15.46 ± 4.55	15.92 ± 6.19	19.32 ± 7.25
Yes	61 (16.71)	67.87 ± 14.51	88.30 ± 15.98	18.46 ± 5.25	18.50 ± 6.90	15.13 ± 4.87	15.78 ± 5.72	20.43 ± 7.47
Time since first cancer diagnosis								
≤1 month	300 (82.2)	67.79 ± 16.52	87.28 ± 16.32	19.36 ± 5.37	17.28 ± 6.79	15.47 ± 4.53	15.68 ± 6.11	19.49 ± 6.95
>1 month	65 (17.8)	69.49 ± 15.34	89.06 ± 16.09	18.45 ± 6.32	19.05 ± 6.92	15.09 ± 4.89	16.90 ± 6.03	19.57 ± 8.73
Perfusion chemotherapy								
No	157 (43)	67.97 ± 15.96	87.99 ± 16.27	18.98 ± 5.54	17.77 ± 7.12	15.52 ± 4.33	15.70 ± 6.02	20.02 ± 6.82
Yes	208 (57)	68.19 ± 16.61	87.30 ± 16.31	19.36 ± 5.57	17.46 ± 6.63	15.31 ± 4.79	16.04 ± 6.18	19.12 ± 7.61
Cancer stages								
I	233 (63.8)	68.57 ± 16.62	88.08 ± 16.42	19.53 ± 5.57	17.62 ± 6.75	15.31 ± 4.61	16.10 ± 6.06	19.51 ± 7.28
II+III	127 (36.8)	67.24 ± 15.76	86.73 ± 16.03	18.59 ± 5.49	17.54 ± 7.01	15.58 ± 4.57	15.53 ± 6.20	19.49 ± 7.33
Histopathological grading								
G1	235 (64.4)	68.44 ± 46.56	87.81 ± 16.67	19.65 ± 5.30*	17.58 ± 6.76	15.31 ± 4.64	15.89 ± 6.18	19.37 ± 7.06
G2+G3	130 (35.6)	67.49 ± 15.89	87.23 ± 15.62	18.40 ± 5.91*	17.61 ± 7.00	15.56 ± 4.53	15.92 ± 6.01	19.74 ± 7.69
Pathologic features								
Urothelial Carcinoma	354 (96.99)	68.04 ± 16.25	87.53 ± 16.15	19.18 ± 5.59	17.57 ± 6.83	15.37 ± 4.60	15.91 ± 6.05	19.49 ± 7.30
Adenocarcinoma	5 (1.37)	65.20 ± 5.71	89.47 ± 11.85	19.20 ± 3.35	15.00 ± 7.73	17.00 ± 3.24	14.00 ± 4.64	24.27 ± 6.65
Squamous Cell Carcinoma	6 (1.64)	73.66 ± 25.57	89.92 ± 27.27	20.33 ± 5.57	20.82 ± 6.55	15.83 ± 5.56	16.67 ± 10.65	16.27 ± 5.86

*SD* standard deviations, *PWB* physical well-being, *SWB* social/familial well-being, *EWB* emotional well-being, *FWB* functional well-being, *BSS* bladder cancer specific questions

*p <0.05, **p <0.01

In Table 1, different levels of the overall QOL and its dimensions were also shown. Participants aged over 75 years had significantly lower scores of QOL. Participants with higher education had higher levels of the overall QOL, SWB, EWB and FWB. Results also indicated that participants exercised regularly had higher level of QOL. Participants with chronic diseases reported lower PWB, and patients with G1 had higher PWB. Differences in other groups were not statistically significant (*p* >0.05).

The mean scores of FACT-BL, social support, hope and resilience were provided in Table 2. Mean (SD) score of FACT-BL was 87.60 (16.27), and the total scores ranged from 49.00 to 129.80. Mean (SD) scores of social support, hope and resilience were 57.55(17.45), 21.55 (4.77) and 65.59 (18.26), respectively.

**Table 2:** Means, standard deviations (SD) and correlations of continuous variables

Variables	Mean	SD	1	2	3	4	5	6	7	8	9
1.PWB	19.20	5.56	1								
2.SWB	17.59	6.84	0.006	1							
3.EWB	15.40	4.60	.550**	.233**	1						
4.FWB	15.90	6.11	.203**	.565**	.459**	1					
5.BSS	19.51	7.29	-.452**	0.045	-.379**	0.037	1				
6.FACT-BL	87.60	16.27	.373**	.720**	.571**	.828**	.219**	1			
7.social support	57.55	17.45	.121^*^	.619**	.208**	.419**	−0.076	.483**	1		
8.hope	21.55	4.77	.212**	.417**	.435**	.504**	-.159**	.489**	.407**	1	
9.resilience	65.59	18.26	.214**	.462**	.402**	.486**	-.149**	.496**	.517**	.687**	1

*SD* standard deviations, *PWB* physical well-being, *SWB* social/familial well-being, *EWB* emotional well-being, *FWB* functional well-being, *BSS* bladder cancer specific questions

*p <0.05, **p <0.01

### Correlation between positive resources and QOL

Pearson’s correlation coefficients were calculated between social support, hope, resilience, FACT-BL and its components. As shown in Table 2, all correlations between FACT-BL, social support, hope, and resilience were statistically significant. Social support, hope and resilience also significantly associated with each dimension of FACT-BL except for the correlation between BSS and social support.

### Hierarchical regression analyses

The indictors of FACT-BL were presented in Table 3. Multiple linear regression analyses were conducted to explore independent variables predicting QOL. Each step of independent variables made a significant contribution to the variance of QOL. Demographic characteristics including age, education level and physical activity as a whole accounted for 5.4 % variance of QOL. After controlling for demographic characteristics, social support, hope and resilience were positively associated with QOL. The three independent variables collectively accounted for an additional 30.3 % variance of QOL. The test of R^2^-change was significant (*p* <0.01), and it indicated that the three influence factors as an integral were the significant predictors of QOL.

**Table 3:** Hierarchical linear regression analysis results, with QOL as the dependent variable

Variables	Step 1		Step 2	
	β	*P*	β	*P*
Control variables				
Age	−0.074	0.153	−0.026	0.552
Edu1	0.059	0.273	0.05	0.262
Edu2	0.182	0.001	0.065	0.150
Physical activity	0.122	0.019	0.034	0.430
Influence factors				
Social support			0.285	0.000
Hope			0.243	0.000
Resilience			0.164	0.010
F	5.098**		28.234**	
R^2^	0.054		0.356	
Adjusted R^2^	0.043		0.344	
△R^2^	0.054		0.303	

*p <0.05, **p <0.01

Edu1 means “Primary/Middle school” vs. “High or secondary school”, Edu2 means “Junior College and over” vs. “High or secondary school”; △R^2^ = R^2^-change

In the models of QOL, tolerance (range: 0.455–0.993) and variance inflation (range: 1.007–2.196) did not indicate a multicollinearity problem.

## Discussion

The results from this study indicated that most Chinese bladder cancer patients suffer from impaired QOL. Mean score of overall QOL value was much lower than that of patients with bladder cancer in developed countries. Yuh et al. indicated that in America, mean score of FACT-G was 84.2, and of FACT-BL was 101.7 [26]. In Mastuda’s research, median of FACT-G was 81.0, and of FACT-BL was 116.8 [2]. In Asian countries like Japan, Kikuchi reported that mean score of FACT-G was 82.0, and of FACT-BL was 107.6 [19]. By contrast, QOL of Chinese bladder cancer patients in our study were at a low level, and there might be four reasons for this circumstance. Firstly, although the morbidity of bladder cancer showed a rising trend in recent decade, China still belonged to one of the moderate level countries in the morbidity of bladder cancer [1]. Meanwhile, less cancer metastasis and high survival rate after surgery resulted in the lack of attention to this disease. Secondly, medical security system was incomplete, which resulted in some patients was not able to get the corresponding treatment measures in time. At the same time, the high recurrence rate of bladder cancer was also increased the burden of hospitalized for patients, resulting in a decline in QOL. Thirdly, psychological disorders of Chinese bladder cancer patients were serious. Surgery and relevant changes (changes in self-image and sexual function) brought individual huge damage of physiology, psychology and social-relation [27–31]. In western countries, developed several clinical practice guidelines for the psychotherapy and supportive care were widely used to cancer patients [32]. But in China, existing medical institutions could not offer professional counseling for cancer patients. Finally, due to the culture of filial piety and the Confucianism spirit in China, family members usually undertook the responsibilities of caring for patients, which could result in great financial and caregiving burden [33]. The only-child policy made burden of the whole family of cancer patient increasingly heavier. These situations collectively exacerbated serious negative impact on cancer patients and caused poor QOL of bladder cancer patients.

### Effects of demographic and clinical variables on QOL and its components

This current study was provided to support that physical activity and education level contributed to enhance QOL, but patients over the age of 75 had worst QOL. Due to the most common physical activity forms of Chinese residents were group activities such as walking outdoor, it contributed to strengthen communication with partners (family or friends) and develop the affection between family and friends. It also conduced to improve their physical health and the overall QOL [34]. Findings from our study also indicated that higher degree graduates had higher level of SWB, EWB and FWB, respectively. In China, owning higher education level means individual could have better job, income, and even higher level of social status. So higher education level individual might have a more rational cognition on real living environments, and had stronger self-adjustment ability. As a result patients with higher education experienced better recovery of psychological well-being and had fewer problems in daily living and work, and negative emotion over time [35, 36]. Yet we found out that older patients had lower QOL. The posttreatment burden of the cancer, such as loss of physical function, fatigue, insomnia, depression, anxiety and economic loss affected disease progression and recovery of elderly patients to a great extent [30, 31]. Older patients had weaker fighting spirit than younger [31]. And likewise, compared with younger patients, patients over the age of 75 faced up with more psychological issues and poor prognosis. All these negative situations might be main reasons for low level of QOL for bladder cancer patients in this study.

### Social support, hope and resilience predicting QOL

The important result of the current study was social support, hope, and resilience were crucial factors to QOL. Social support, hope and resilience, as a whole, positively connected with QOL. Most importantly, the two aspect (external and internal) influence factors jointly explained 30.3 % variance of the overall QOL, which had the stronger predictive values than each of them (data was not shown), indicating that the integrative measure of social support, hope and resilience may be more realistic and effective than using the single construct to predict QOL. Cancer as a serious life-threatening illness made negative effects on QOL of patients. Patients with cancer faced great psychological pressure, and significantly higher depression and anxiety in China [37]. These passive emotions impaired both physical and psychological health of patients. In line with previous studies, positive psychosocial factors influenced the appraisal of stressful situations and optimize QOL [33, 37]. Positive social and psychological attributes were effectively prevented negative emotions and promoted well-being [38], alleviated pain perception [39], and enhanced QOL of bladder cancer patients [27].

Social support was a predictive factor of QOL. Social support was positively associated with QOL, in agreement with previous research [8, 40]. Lack of social support resulted in maladaptive coping responses to cancer, further to weak fighting spirit of cancer patients [41], and ultimately affected the treatment effect. Likewise, low level of social support might induce higher risk of both incidence and mortality in cancer patients [5]. Wu et al. also found out that social support could relieve the deleterious impact of negative feelings on QOL, such as depression and depressive symptoms [42]. Patients could obtain psychological comfort from family and friends, and solved problems through positive pathways. At the same time, cancer patients might emerge more strongly dependent feelings and psychological safety, which stimulated strong self-confidence. All the favorable results were helpful for clinical treatments and recovery. Therefore, patients with bladder cancer could get benefits from social support, and observed the positive meaning in cancer experience [7]. Hodges and Winstanley demonstrated that social support made positive effect on cancer patients and promoted well-being, and further to improve the overall QOL, particularly mental health [8]. To bladder cancer patients, enough social support was essential to enhance the overall QOL.

As well as social factor, this study also revealed that positive psychological factors (hope and resilience) played a significant role in QOL. Hope has been found to help patients adapt to and give meaning in cancer, maintain a high level of well-being, and give directions and reason for survival [43]. Hope provided individuals a positive resource for combating psychological issues as depression and anxiety while protecting against perceptions of vulnerability and unpredictability [44]. For instance, patients with high level of hope were likely to have fewer mood symptoms, because higher hope people were more likely to engage in positive cancer-related thoughts and leading to more positive outcomes [45]. Hope also had therapeutic value for patients with cancer. Felder et al. investigated that there was positive relationship between hope and treatment efficacy in patients with breast cancer [46]. Pulvers et al. indicated that the higher the level of hope, the more cancer pain a patient could tolerate [39]. Hope played a substantial role in preventing QOL impairment [47], and was also considered as a psychological and spiritual resource which was beneficial in fighting cancer [6]. Higher hope individuals might be more likely to participate in more activities with their spouse to achieve their goal of a better relationship [48]. In other words, hope had beneficial effect on psychological well-being, physical well-being and QOL [48, 49]. The diagnosis of cancer and its subsequent treatment might increase patients’ emotional disturbance and decrease their levels of hope [50], but cancer patients revealed a high hope level in western countries [51]. This could also be the cause of the QOL for bladder cancer patients in China lower than western countries. Thus, effectively improve the level of hope was one of the most important ways to stimulate the QOL of bladder cancer patients in China.

Resilience was also positively related to QOL in this study. Resilience has been confirmed to moderate negative effects of stress and promoted positive adaptation [14]. Strauss et al. emphasized that resilience could effectively reduce complains of cancer patients treated with radiotherapy, and had benefits to the initial fatigue [52]. Because of the high degree resilience of patients had a stronger fighting spirit and firmer belief in fighting against cancer. Therefore patients with high resilience were easier to accept and adjust for psychological damage. Likewise high resilience can help patients reduce treatment-induced damage to physical functions and shorten the time of recovery of physical functions [53]. Resilience, as a positive psychological construct, also played a significant role in treating cancer correctly and promoting favorable psychological health [25], thereby resulted in a better QOL before, during and after cancer [54]. Resilience could be most effectively to best equip cancer patients to avoid negative emotions, such as depression and anxiety disorder [13]. Similarly, resilience could appear at each time point with different clinical characteristics, and it was able to be fostered by various types of interventions or interactions [54]. Patients with greater internal strength had reported reduced distress, better coping strategies, and improved QOL [55]. To bladder cancer patients, resilience was a critical psychological factor to enhance the overall QOL.

## Implications

Several importantly theoretical and practical implications emerged from the findings of this study. In theory, this study provided the preliminary possibility of building a higher-order, core-positive psycho-social construct to enhance QOL in bladder cancer patients by synthesizing and integrating both the external and internal constructs of social support, hope and resilience. In practice, there were three implications. Firstly, the low level of QOL in bladder cancer patients should receive sufficient attention by Chinese medical institutions and government. Secondly, it was important for oncologists and physicians to pay more attention to patients with lower education level. Last and most importantly, a new perspective would be provided for researchers on the use of an integrated model to improve positive psycho-social resources and QOL in cancer patients by synthesizing and integrating the protective effects of social support, hope, and resilience. Some studies have provided the concrete measures and advices to implement psycho-social interventions to enhance QOL in cancer patients, but intervention results were controversial. Baños reported that positive psychological intervention (virtual reality) was available to enhance positive emotions and decrease negative emotions [38]. But Boesen found out that psycho-education and group psychotherapy did not contribute to increase QOL or decrease psychological distress [56]. Further studies should be conducted to prove whether the integrated psycho-social interventions based our findings and other studies are effective in bladder cancer patients as well as any other fields of oncology.

## Limitations

There were several limitations for the present study. Firstly, it characterized by cross-sectional research based on self-reported measures, so one cannot derive any conclusions on the causality of the associations observed between psycho-social resources and QOL. Additionally, the interpretation of the results should be made with caution because we did not include a control group, otherwise, the level of QOL in cancer patients can be more reliably and accurately determined. Furthermore, subjects were from a single hospital in China, which may limit the generalizability of this study to other regions. Lastly, further studies need to be conducted to examine whether the results of the present study are suitable to the different cultural context. These above insufficient points would need to be substantiated in future research.

## Conclusion

In conclusion, our results indicated that QOL of bladder cancer patients was at a low level. More importantly, social support, hope and resilience had a considerable effect on their QOL. This was the first attempt to perform the relationship between positive psycho-social resources and QOL of patients with bladder cancer in China to our knowledge. This present study highly recommended that positive social and psychological resources would be helpful to support QOL of bladder cancer patients. Likewise, the findings supported that QOL of bladder cancer patients should receive more attention from Chinese medical institutions. The present study also put an insight into the integrative effects of social support, hope and resilience in relation to QOL. Targeted support for the bladder cancer patients, such as positively psycho-social interventions might be useful to support their QOL in oncology field.

## Ethical considerations

This study received ethics approval from the Committee on Human Experimentation of China Medical University and the First Hospital of China Medical University (Shenyang, Liaoning, China). Written informed consent concerning conduct of the survey was obtained from each participant. We protected the privacy of individuals in processing personal data and maintained confidentiality of individual records and accounts.
